# Effect of Laser Power on Microstructure and Properties of WC-12Co Composite Coatings Deposited by Laser-Based Directed Energy Deposition

**DOI:** 10.3390/ma17174215

**Published:** 2024-08-26

**Authors:** Wen Li, Husen Yang, Yichun Liu, Fengxian Li, Jianhong Yi, Jürgen Eckert

**Affiliations:** 1 Faculty of Material Science and Engineering, Kunming University of Science and Technology, Kunming 650093, China; lw1186637944@163.com (W.L.); wy1282223420@163.com (H.Y.); spsjtu@163.com (Y.L.); yijianhong@kmust.edu.cn (J.Y.); 2 Erich Schmid Institute of Materials Science, Austrian Academy of Sciences, Jahnstraße 12, A-8700 Leoben, Austria

**Keywords:** DED-LB, composite coating, wear property, laser power

## Abstract

During the laser-based directed energy deposition (DED-LB) processing, a WC-12Co composite coating with high hardness and strong wear resistance was successfully prepared on a 316L stainless steel substrate by adopting a high-precision coaxial powder feeding system using a spherical WC-12Co composite powder, which showed a large number of dendritic carbides and herringbone planar crystals on the substrate-binding interface. The influences of laser power on microstructural and mechanical properties (e.g., hardness, friction resistance) of WC-12Co composite surfaces were investigated. The results show that laser power has a significant effect on determining the degree of Co phase melting around the WC particles and the adhesion strength between the matrix and the coating. Lower laser power does not meet the melting requirements of WC particles, thus weakening the molding quality of the composite coating. At high laser power, it is possible to dissolve the WC particles and melt the metal powder between the particles, thus improving the material properties. The laser power increased from 700 W to 1000 W and the average hardness of the coating surface gradually increased from 1166.33 HV to 1395.70 HV, which is about 4–5 times higher than the average hardness of the substrate (about 281.76 HV). In addition, the coatings deposited at 1000 W showed better wear resistance. This work shows that the processing parameters during laser-directed energy deposition can be optimized to prepare WC-12Co composite coatings with excellent mechanical properties.

## 1. Introduction

Laser-based directed energy deposition (DED-LB) is a prospective additive manufacturing (AM) technology that uses a vigorous laser beam to deposit components with complex geometries, high dimensional accuracy, and high power [1,2]. Metal–ceramic coatings prepared by conventional thermal spraying methods are very susceptible to rusting due to their porous nature, large cracks, and variations in oxide phase types [3]. Compared with other coating preparation techniques, coatings prepared by laser-directed energy deposition have several advantages: a short forming time to minimize strain and deformation in the deposited material; the metallurgical bond between the substrate and the coating material is stronger; faster cooling and finer microstructure, which improves the mechanical strength of the coating, etc. [4]. Kyoung-Wook Kim et al. [5] fabricated WC-12Co cemented carbide materials using the directed energy deposition (DED) process and compared their wear characteristics with those of WC-12Co cemented carbide materials fabricated by the high-velocity oxygen fuel (HVOF) spraying process and found that the DED samples had the lowest wear rate and the best wear resistance. DED can be categorized into DED-LB and wire arc DED, which can also be called wire arc additive manufacturing (WAAM), depending on the feedstock used [6]. Compared with powder-based DED-LB, the wire feedstock used in WAAM is three to five times cheaper than powder of the same material. Moreover, WAAM improves the material utilization efficiency, and the use of powder as a feedstock disperses in the melt pool, sometimes leaving unmelted particles on the part surface or substrate [7]. However, WAAM tends to accumulate a large amount of melted wire at the corners, especially if the corners are sharp. Therefore, WAAM is more suitable for single-pass coating melting, and it may have large dimensional deviations during the actual construction process, which accumulate layer-by-layer, greatly reducing the dimensional accuracy of the components and hindering the completion of stacking according to the planned path [8]. During the molding process, the thermal state of the material is very complex due to the heat source moving back and forth on the previous layer [9], resulting in defects in the final coating dominated by holes, cracks, surface roughness, and residual stresses [10]. In order to fabricate well-bonded 24 × 24 mm WC-12Co composite coatings, we chose to use DED-LB.

Nowadays, there is a growing demand for new coating materials to meet the needs of extreme working environments [11]; thus, more and more people are turning their attention to the processing of advanced materials such as high-entropy alloys (HEAs), functionally gradient materials (FGMs), or metal matrix composites (MMCs) with additive manufacturing (AM), among which WC-Co composite coatings have gained a lot of attention.

WC-Co carbides consist of a wear-resistant WC hard phase and a ductile Co-bonded phase [12], where the Co-bonded phase is typically under 25%. They are known as the “teeth of industry” because of their high hardness, wear resistance, compressive resistance, and elastic modulus [13]. It is widely used as a coating material to improve the quality of surfaces of other metals [14,15]. In this paper, the spherical WC-12Co powder is used as materials, employing a laser energy deposition coaxial powder spraying process on 316L structural steel for the preparation of WC-12Co composite coatings. However, WC-Co composite coatings prepared by rapid prototyping are prone to defects such as porosity, cracks, and excessive thermal stresses due to large temperature gradients, brief heating and solidifying times, and large differences in the physicochemical properties of the metals of the WC hard phase and the Co-bonded phase. There is a lot of literature on the reduction in defects in WC-Co composite coatings. Mohammad et al. [16] attempted to relate the key process parameters to the geometry and porosity of the single cladding layer in order to minimize the defects in laser melted WC-12Co powder on the surface of AISI 321 steel; Son et al. [17] found that the mechanical properties and crack extension behavior of selective laser melting (SLM) formed WC-Co cemented carbide were affected by the microstructure inhomogeneity of WC particles; Farahmand et al. [18] produced uniform and crackless composite coatings by adding La_2_O_3_ and investigated the influence of rare earths on the quality of the coatings during laser melting.

The WC-12Co spherical composite powder used in this paper has high sphericity, a smooth surface, and good fluidity, which is conducive to the uniform distribution of the hard phase WC in the coating. The prepared WC-12Co composite coatings remarkably enhanced the hardness and resistance to wear of the substrate. The hardness and wear resistance of the substrate were enhanced significantly by the preparation of WC-12Co composite coatings. The defects in the coatings were reduced by changing the technological parameters, and the relationship between the microstructural and mechanical properties of the WC-12Co composite coatings and the effect of changing the laser power on the microstructural and mechanical properties were investigated.

## 2. Experimental Section

### 2.1. Materials

An AISI 316L stainless steel plate with a size of 100 × 100 × 5 mm was used as the substrate, and in order to avoid the influence of impurities on the substrate, the surface of the substrate was polished using sandpaper and ultrasonically cleaned prior to the deposition of laser-directed energy. Table 1 lists the chemical composition of the substrates. WC-12Co-coated powder (produced by Guangzhou Metal Metallurgy Company, Guangzhou, China) with a particle size distribution in the range of 15–45 μm was used for coating deposition.

### 2.2. Experimental Processes

**Figure 1:**
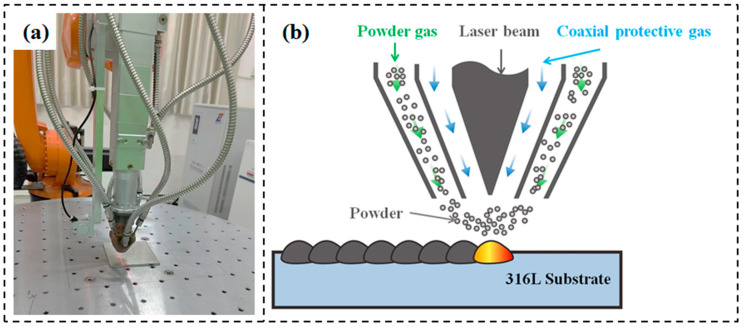
(**a**) Diagram of the DED-LB experimental set-up. (**b**) DED-LB process principle schematic.

In this study, WC-12Co composite coatings were deposited by using the DED-LB system (Digilight-4000, Kunming University of Science and Technology, Kunming, China). The mixed powders were delivered to the printing nozzle by using a high-precision and intelligent coaxial powder feeding system under an argon atmosphere protection and a gas pressure of 0.5 MPa, as shown in Figure 1. Then, the mixed powders were deposited on the substrate according to the designed scanning strategy at a powder flow rate of 18 g/min. The laser system has a wavelength range of 1060–1100 nm and a peak power of 4000 W. In order to understand the effect of laser power on the microstructure and mechanical properties, the laser power was set to 700 W, 850 W, and 1000 W, while other parameters were fixed, such as scanning speed of 20 mm/s and spot diameter of 1.6 mm.

Too low a laser power results in failure of WC particles to melt, while laser power above 1000 W results in evaporation of the cobalt binder. Paul et al. [19] reported that in order to obtain high-quality dense WC-12Co composite coatings, the average energy density per unit area and the maximum power density per unit area should be about approximately 30 Jmm^−2^ and 350–600 Wmm^−2^. The processing parameters setup in this study satisfies the conditions reported by Paul et al. [19].

**Figure 2:**
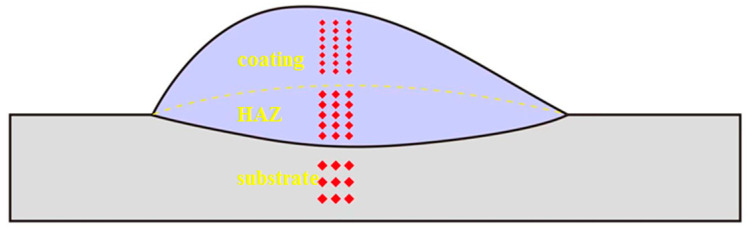
Schematic diagram of the area of cross-section hardness measurement.

For the characterization of microstructures and phases, a scanning electron microscope equipped with an energy-dispersive spectrometer (EDS) (Sigma 300, Carl Zeiss AG, Jena, Germany) (SEM, Sigma 300, 20 kV) and an X-ray diffractometer equipped with a Cu target and Ka radiation (Mini Flex 600, Rigaku Corporation, Tokyo, Japan) (XRD; Rigaku, Cu-Kα, 2θ = 10–90°, rate = 5°/min) were used. Microhardness measurements were performed on a Vickers hardness tester (HVS-1000Z) with an applied load of 9.8 N for 15 s. The microhardness was measured 15 times at equal distances along the longitudinal length of the cross-section: 7 measurements in the coating area above the bond line, 5 measurements in the heat-affected zone (HAZ) at the bond, and finally, 3 measurements at the substrate location below the bond line. The specific hardness test areas are shown schematically in Figure 2. To minimize random errors, three hardness measurements were taken at points of the same height and averaged. Friction and wear tests were carried out using a multifunctional rotary friction tester (MS-M9000) with a friction pair of 5 mm diameter high hardness alumina ceramic balls, the rotational speed of the turntable set at 200 r/min, an applied load of 15 N, a rotational radius of 5 mm, and a loading time of 60 min.

## 3. Results

### 3.1. Microstructure Evolutions

**Figure 3:**
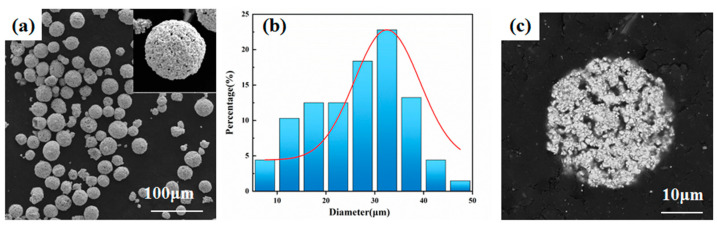

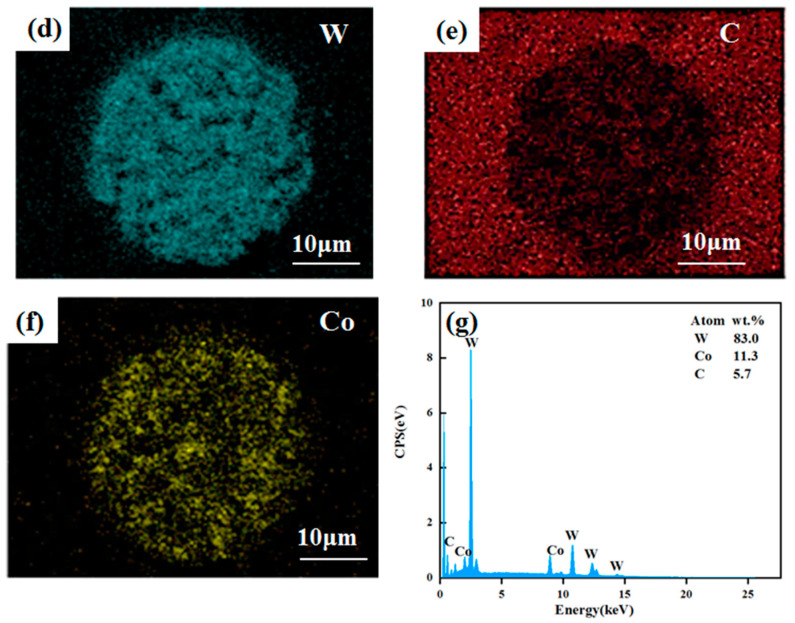
(**a**) SEM image of WC-12Co coated powder. (**b**) Corresponding powder particle size distribution. (**c**) Cross-section of coated powder and (**d**–**g**) EDS elemental analysis of cross-section.

A scanning electron microscope was used to characterize the morphology of the raw material, and energy-dispersive spectroscopy (EDS) was performed to analyze the powder cross-section. The morphology of the powder used for laser-directed energy deposition is shown in Figure 3. As can be seen from Figure 3a, the WC-12Co coated powder has a high sphericity and uniform particle size, which indicates that it has good fluidity, which is favorable for the laser-directed energy deposition of the coating with a uniform distribution of the WC hard phases. The cross-section in Figure 3c shows the angstroms in the cladding powder, and the elemental content of the cladding powder can be seen in Figure 3d–g.

**Figure 4:**
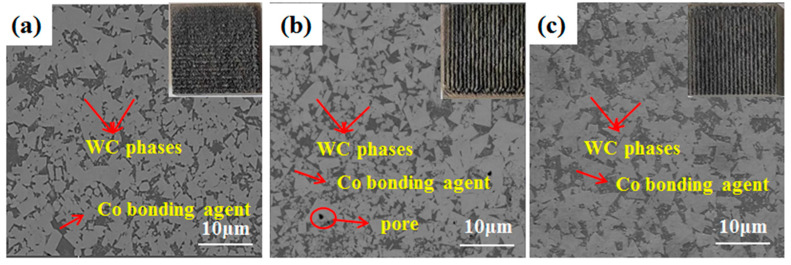
Scanning electron micrographs of composite coatings at different power levels: (**a**) 750 W, (**b**) 850 W, (**c**) 1000 W.

The change in laser power changes the coating surface appearance, and Figure 4 shows the surface appearance of the coatings at different powers. The surface of the WC-12Co composite coating deposited at 700 W is the roughest with a large amount of residual powder, which is due to the low laser power, and a large amount of coaxially fed powder is not completely melted on the surface of the coating. This situation improves with a gradual increase in power, and the surface becomes smoother and has less residue.

**Figure 5:**
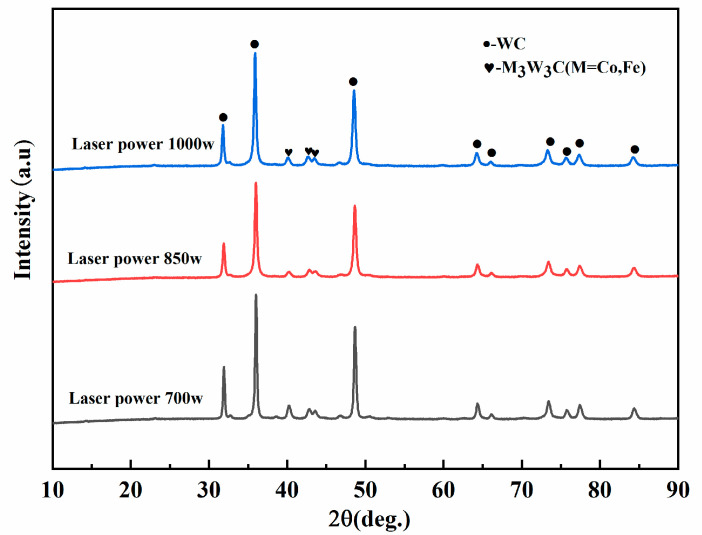
XRD spectra of WC-12Co composite coatings at different laser powers.

An X-ray diffraction analysis was performed on the surface of the WC-12Co composite coating samples to investigate the phase composition and its effects on the microstructure of the composite coating. Figure 5 displays the X-ray diffraction spectrum of the WC-12Co composite coating surface at different laser powers (700 W, 850 W, and 1000 W). It can also be seen that the WC-12Co composite coating is mainly composed of WC and M_3_W_3_C (M = Fe,Co) amorphous carbides. Although the laser power was increased from 700 W to 1000 W, there was still no completely melted WC particle residual phase on the surface of the coating. When the laser power was increased from 700 W to 850 W, the diffraction peaks of the WC residual phase gradually decreased, and when the laser power was increased to 1000 W, the phase diffraction peaks of the WC phase increased again. The intensity and width of the diffraction peaks of the WC phase changed negatively with the increase in the laser beam power. The intensity of the diffraction peaks became weaker and the width of the peaks became larger. There was no discovery of secondary carbides such as W_2_C or WC decarburization in the XRD spectra of the coatings, and it was presumed that the specific reaction should be carried out inside the coating, and the nascent phase could not be seen on the surface of the coating. The diffraction peak strength of M_3_W_3_C (M = Fe,Co) amorphous carbide is opposite to that of WC, which is due to the spreading of W and C atoms from the dissolution of WC particles into the AISI 316L stainless steel matrix after thermal impact by a high-energy-density laser. At the same time, the Fe and C elements in 316L diffuse into the WC particles to form new M_3_W_3_C (M = Fe,Co) eutectic carbides. As a result, the M_3_W_3_C phase increases while the WC phase decreases [20]. The diffraction peak of eutectic carbide is highest at 700 W. The specific phase pattern of eutectic carbide generation is analyzed in the following section in conjunction with SEM images.

### 3.2. Hardness

**Figure 6:**
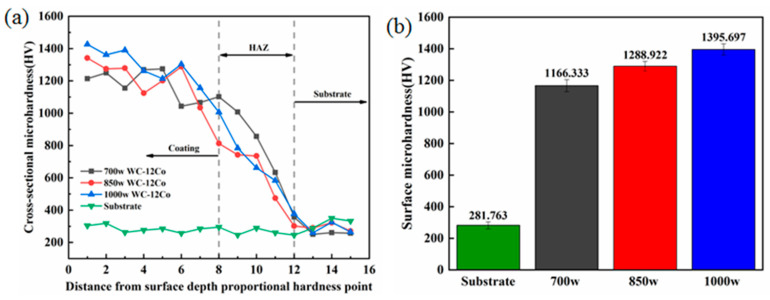
Microhardness distribution of coatings under different laser powers: (**a**) variation of hardness of coating cross-section under different powers, (**b**) average hardness of coating surface under different powers.

The hardness of the cross-section and all areas of the coated surface varies with hardness. Figure 6 displays the microhardness distribution of the WC-12Co composite coating in the cross-region, and it can be seen that the WC-12Co composite coating can obviously enhance the surface hardness of the 316L stainless steel substrate. At 700 W, 850 W, and 1000 W laser power, the average hardness of the coating surface was 1166.33 HV, 1288.92 HV, and 1395.70 HV, respectively, which was significantly higher than the average hardness of the substrate (about 281.76 HV). It can be seen that the average hardness of the coating is improved with the increase in laser power. Lower laser power results in lower input laser power as well, which leads to insufficient growth of WC and M_3_W_3_C (M = Fe,Co) grains, thereby resulting in lower hardness of the coating [21]. 

From the hardness changes in the coating cross-section in Figure 6a, it can be seen that the microhardness profile from the composite coating to the base body generally shows a gradual decrease in the microhardness distribution curve. In the coating area, hardness is above 1000 HV; in the heat-affected zone (HAZ), hardness gradually reduces to 800 HV, 600 HV, 400 HV or so, until it reduces to the hardness of the substrate to maintain consistency. This is the composite coating and the substrate combined with a good, smooth transition embodying the composite coating to avoid the composite coating in the combination of the thermal stress impacting cracks or even falling off. Overall, WC is more uniformly distributed and better metallurgically bonded, resulting in higher microhardness [22,23]. With the increase in laser power, the high-energy-density laser produces high heat to melt the powder, creating a longer molten pool, allowing the carbide in the molten pool to fully grow and flow, resulting in a smaller WC phase size and finer grain size.

### 3.3. Wear Resistance

**Figure 7:**
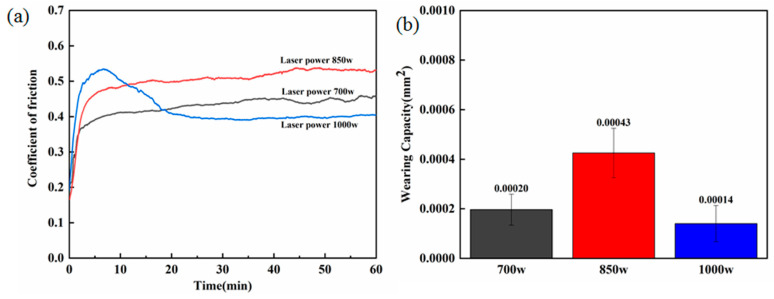
Friction wear of coatings at different laser powers: (**a**) friction coefficient, (**b**) average wear.

To research the impact of laser targeted energy deposition on the tribological properties of WC-12Co composite surfaces at different laser powers, friction-induced creep tests were conducted on WC-12Co composite surface samples. As shown in Figure 7a, the rule of friction coefficient variation with sliding time was obtained by recording the friction coefficient variation of the samples during the frictional wear process. It can be seen that the laser power plays a key role in the wear characteristics.

When the laser power is 700 W, the average coefficient of friction (COF) is 0.426 and the average wear amount lost is 0.00020 mm^2^. The lower laser power leads to a lower energy density as well, and the presence of unmelted WC particles brings about a high wear amount. When the laser power is 850 W, the curve rises and the average COF increases to 0.499, and the abrasion loss is also the highest at 0.00043 mm^2^. As the coating adheres poorly to the substrate at 850 W, the WC particles are gathered at the bonding interface, which produces hole-cracking defects, reducing the bonding of the coating, and the large number of wear-resistant WC phases are not uniform, which decreases the wear-resistant property of the samples. As the laser power rises to 1000 W, the curve is divided into two periods: the break-in period and the steady wear period [24]. The COF increases rapidly to a maximum value of 0.535 when the friction pair and the coating first come into contact; this is due to the initial break-in phase, where the interfacial film is abraded due to the pressure, creating a cold weld effect at the point of contact, which requires high shear to cut the weld joints [25]. As the friction time increases, the friction vice and the coating stabilize, resulting in grinding and the formation of a certain oxide layer lubrication; the COF gradually decreases and levels off after 20 min.

## 4. Discussions

### 4.1. Evolution of Pores and the Bounding Strength

**Figure 8:**
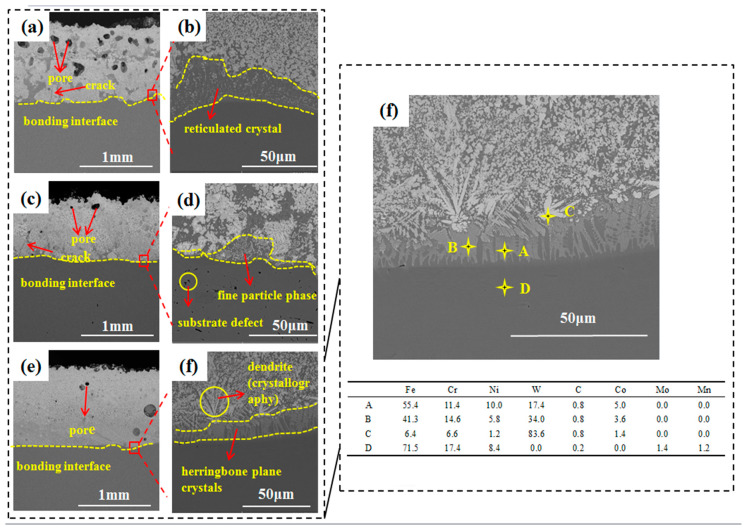
SEM images of the cross-section of WC-12Co composite surfaces at different laser powers: (**a**,**b**) 700 W, (**c**,**d**) 850 W, (**e**,**f**) 1000 W.

Adjusting the laser power can reduce the defects of the surface composite coatings. Figure 8a,c,e show the macroscopic images of the coating cross-regions when the laser power is 700 W, 850 W, and 1000 W, respectively, and it can be clearly seen that there are many large-size air holes at the top of the coating and a mesh crystal transition at the combination interface when the laser power is 700 W. When the laser power is increased, the size and number of the air holes decrease. When the laser power increases, the size and number of pores gradually decrease. When the laser power is 850 W, although the pores of the composite coating are reduced, the cracks in the middle are the deepest, the distribution of the WC phase is extremely uneven, with no transition crystals at the bonding interface, and there are many defects on the substrate due to thermal stresses. When the laser power is raised to 1000 W, the WC-12Co composite coating has only slightly smaller pores, no cracks, and is densely bonded. This is because, as shown in Figure 8f, when the laser power is increased to 1000 W, directionally developed dendritic carbides are produced at the bonding interface and finely tuned enhancement particles are uniformly dispersed in these dendritic carbides. A uniformly flat herringbone-shaped eutectic carbide transition between the dendritic carbide phase and the substrate also occurs; as a result, the bond of the coating to the substrate is stronger and performs better. In Figure 8f, different parts of the black substrate site are dotted swept, and the role of the flat herringbone eutectic carbide transition can be seen from the Fe content.

### 4.2. The Inhibition of Crack

In general, when other process parameters are fixed, the thickness of the deposited layer increases with increasing laser power [26], which is usually due to the change in the surface energy density as a result of the change in laser power. This is mainly characterized by the representation of the beam energy parameter responsible for the melting of the powder and substrate surfaces, which can be described by the following equation [27]:

(1) $$E=\frac{P}{VD}$$

where V is the scanning speed of the laser head (mm/s), D is the diameter of the laser spot (mm), and *P* is the laser power (W). In the laser deposition process, the energy density plays a decisive role in the height of the deposited layer. The higher the energy of the laser, the greater the deposition height because the higher the energy density, the higher the heat content and the more efficient the melting of the powder [28].

Figure 8a shows that at 700 W, the deposited coating height is 0.93 mm; at 850 W, the deposited coating height is 0.77 mm; and at 1000 W, the deposited coating height is 0.99 mm. Obviously, 850 W does not match the trend, as the coating height is greatly reduced. From Figure 8d, it can be seen that at a laser power of 850 W, the coating and the substrate are not close enough, the white prismatic WC phase is large and small, and the distribution is not uniform, which may be the main cause of the reduction in the deposited layer. Additionally, the distribution is not uniform, and there are obvious cracks, which may be the main reason for the reduction in the deposited layer.

**Figure 9:**
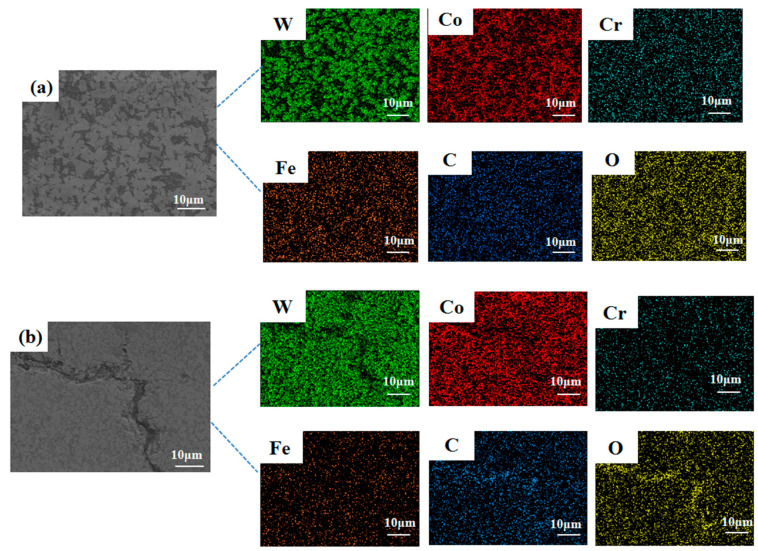
SEM images of composite coatings: (**a**) coating at 1000 W, (**b**) coating with cracks at 850 W.

Zhou et al. [29] showed that the number and behavior of cracks mainly depend on the porosity, distribution characteristics, WC dissolution, and residual stresses of the composite coating. As can be seen in Figure 9, the cracks in the cross-section of the 850 W coating are enriched with C and O elements compared to the coating without cracks. This may be caused by the solubilization and decarburization of the WC in the WC-12Co powder by the laser. The following four phases were observed in the investigated melt baths [30]: decomposition of WC, decarbonization of W_2_C, generation of gases, and generation of M_3_W_3_C:

(2) $$2WC\to W_{2}C+C$$

(3) $$W_{2}C\to 2W+C$$

(4) C+O→CO

(5) $$WC+2W+3M\to M_{3}W_{3}C$$

Although high purity argon gas of 99.999% is used for protection during the deposition of the coating, atmospheric oxygen still enters the molten pool during the rapid heating process of the laser. As shown in Equations (2) and (3), the formed C-elements then combine with the O-elements in the molten bath to form CO gas and even CO_2_ gas in the monoclinic state in the presence of sufficient oxygen. In the subsequent rapid solidification process, gas bubbles are released into the atmosphere after the WC-12Co coating solidifies, causing microbubbles to be entrapped in the coating [31] and forming micropores, as shown in Figure 8a,c,e. When the combination of micropores and Co bonding expands, cracks are formed, which have an impact on the mechanical properties of the coating.

**Figure 10:**
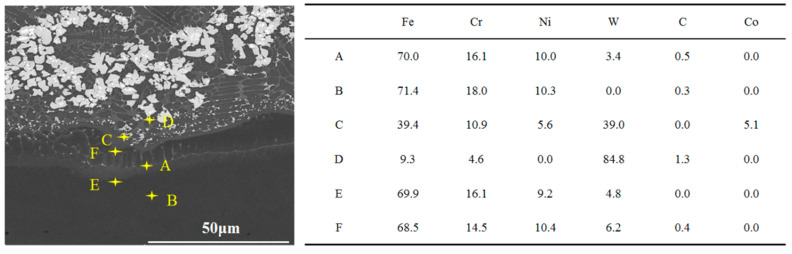
BSE image of WC-12Co composite coating when the laser power is 700 W and the corresponding EDS mapping results.

As we approach the interfacial bonding region, the uniformity of the WC particle distribution disappears, and the size of the WC phase changes. As shown in Figure 10, the matrix becomes very complicated, enriched with columnar carbides and complex eutectic carbides [32], which has been well explained by many authors [20,33]. As the WC particles are dissolved in the matrix, the steel alloying elements in the 316L stainless steel matrix and the faster cooling rate of DED-LB promote a large amount of eutectic carbide precipitation at the bonding interface. Figure 10 displays an SEM picture of a typical area at the bottom of the WC-12Co composite coating at 700 W laser power. They were analyzed using an energy spectrometer, and the black matrix areas (points A and B); white areas (points C and D); and gray, bonded, heat-affected areas (points E and F) were obtained. From the elemental content table, it can be seen that although the elemental content of Fe is close to that of point AB in the same black matrix region, point A has 3.4% more W than point B, which is enough to prove that the region where point A is located is the binding heat-affected zone (HAZ). Only point C was detected with Co element, proving that the gray-white particles on the white area hit by EDS are the bonded Co phase in the original WC-12Co powder, while the EF point on the gray area is consistent with the M_3_W_3_C elemental distribution.

### 4.3. Wear Resistance Mechanism

**Figure 11:**
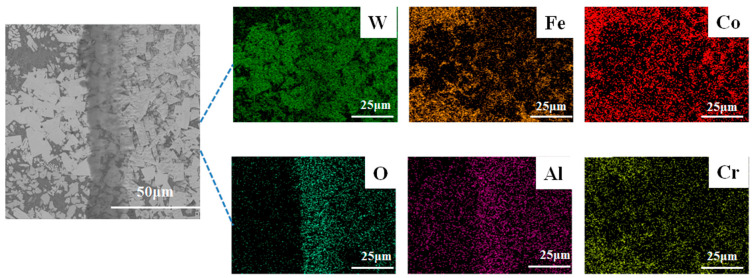
SEM and EDS images of wear tracks of 1000 W laser power WC-12Co coating.

Figure 11 shows the SEM image of the wear pattern of the WC-12Co coating at 1000 W laser power. It can be seen that the wear surface is essentially flat and smooth, which indicates a high wear resistance of the WC-12Co coating. In order to further investigate the wear mechanism of the WC-12Co wear-resistant coating, the chemical element distribution of the wear tracks was analyzed via EDS surface scanning. The presence of friction sub-elements (Al and O) and the precipitation of WC particles on the surface in EDS analyses indicate the presence of abrasive wear mechanisms during the wear process [22,34]. Moreover, the wear track contains a large amount of O elements, and the mass fraction of O at the wear marks is significantly higher than that of the unworn coating, and the exposed wear surface reacts oxidatively with O [35], and forms an oxidized layer at the surface of the WC-12Co wear-resistant coatings, which indicates that the main wear mechanism is oxidative wear. In summary, the average coefficient of friction and the amount of wear of the WC-12Co coating are the lowest at 1000 W laser power, when the coating has the best wear resistance.

## 5. Conclusions

WC-12Co composite coatings with high hardness and strong abrasion resistance have been successfully prepared by using a high-precision coaxial powder delivery system in a DED-LB process. The influence of laser power on the microstructure evolution and mechanical characterization (i.e., hardness, friction wear resistance) of the WC-12Co composite coatings was researched and the following observations were found:

(1) WC-12Co composite coatings prepared by adopting a high-precision coaxial powder feeding system during the DED-LB processing revealed a large number of dendritic carbides and herringbone plane crystals at the matrix bonding interface.
(2) With the increase in laser power from 700 W to 1000 W, the average hardness of the coating surface gradually increased from 1166.33 HV to 1395.70 HV, which was about 4–5 times of the average hardness of the substrate (about 281.76 HV). Moreover, the coatings deposited at 1000 W showed better wear resistance.
(3) The laser power has a significant effect on the energy input, which determines the melting extent of the Co phases around WC particles and the bonding strength between the substrate and the coatings. Adjusting the laser power can significantly reduce the pores in the WC-12Co composite coating, promote the formation of a large number of dendritic carbides and herringbone plane crystals at the matrix bonding interface, improve the bonding strength between the coatings and the substrate, make the WC phase distribution more uniform, and refine the grains, so as to achieve the effect of improving the mechanical properties.

This work suggests that the processing parameters during laser-directed energy deposition can be optimized to prepare WC-12Co composite coatings with excellent mechanical properties.

## Figures and Tables

**Table 1:** Chemical compositions of AISI 316L.

Elements	Fe	Cr	Ni	P	S	Mn	Si	Mo	N	C
Percent (wt.%)	Bal.	16.080	10.090	0.027	0.005	1.150	0.450	2.030	0.048	0.015

## Data Availability

The raw data supporting the conclusions of this article will be made available by the authors on request.
